# Antibiotics influence the risk of anti-drug antibody formation during anti-TNF therapy in Chinese inflammatory bowel disease patients

**DOI:** 10.3389/fphar.2024.1360835

**Published:** 2024-04-09

**Authors:** Meng Sun, Jingyi Ju, Hongzhen Xu, Mengqi Luo, Zhaoyang Li, Yufang Wang

**Affiliations:** Department of Gastroenterology and Hepatology, West China Hospital, Sichuan University, Chengdu, China

**Keywords:** anti-drug antibodies (ADAs), anti-tumor necrosis factor (anti-TNF), infliximab, adalimumab, serum drug concentration, antibiotics, inflammatory bowel disease

## Abstract

**Aims:** The formation of anti-drug antibodies (ADAs) during anti-tumor necrosis factor (anti-TNF) therapy is reported to lead to reducing serum drug levels, which may bring about a loss of response to treatment. Previous research has suggested an association between specific antibiotic classes and ADA formation during anti-TNF therapy. However, there are few studies specifically examining this association in Chinese inflammatory bowel disease (IBD) patients. Therefore, our study aimed to evaluate the possible effect of antibiotic use on ADA formation to anti-TNF therapy in Chinese patients with IBD.

**Methods:** A total of 166 patients with IBD, including 149 with Crohn’s disease (CD) and 17 with ulcerative colitis (UC), were included in this retrospective analysis. These patients were initially treated with anti-TNF therapy (infliximab or adalimumab) after January 2018 and reviewed with available ADA levels before October 2023. After univariable analysis of all the variables, a multivariate Cox proportional hazards model was used to assess the association between antibiotic use and ADA development.

**Results:** Among 166 IBD patients treated with infliximab (108/166, 65.1%) or adalimumab (58/166, 34.9%), 31 patients (18.7%) were measured as positive ADA levels. Cox proportional hazard model demonstrated an increased risk of ADA formation in IBD patients who used β-lactam-β-lactamase inhibitor combinations (BL-BLIs) (HR = 5.143, 95%CI 1.136–23.270, *p* = 0.033), or nitroimidazoles (HR = 4.635, 95%CI 1.641–13.089, *p* = 0.004) during 12 months before the ADA test. On the contrary, a reduced risk was noted in patients treated with fluoroquinolones (HR = 0.258, 95% CI 0.072–0.924, *p* = 0.037). Moreover, the median serum infliximab or adalimumab concentration in patients with positive ADA levels was significantly lower than that in patients with negative ADA levels (infliximab: 0.30 vs. 1.85 μg/mL, *p* < 0.0001; adalimumab: 0.45 vs. 7.55 μg/mL, *p* = 0.0121).

**Conclusion:** ADA development is associated with various antibiotic classes. BL-BLIs and nitroimidazoles might increase the risk of ADA formation during anti-TNF therapy in Chinese IBD patients, while the treatment with fluoroquinolones could probably reduce such risk. There were certain limitations in the retrospective analysis of the study, therefore, the results are just for reference, and other studies are needed to further confirm our findings.

## 1 Introduction

Inflammatory bowel disease (IBD), encompassing ulcerative colitis (UC) and Crohn’s disease (CD), is a chronic and relapsing immune-mediated disease of the gastrointestinal tract, which can lead to a debilitating condition without effective treatment. Currently, the medical therapies for IBD mainly include 5-aminosalicylic acid (5-ASA) (such as mesalamine and sulfasalazine), corticosteroids (such as budesonide and prednisone), immunosuppressants (such as azathioprine and methotrexate) and biologic agents (e.g., anti-TNF, anti-integrins, anti-interleukin-12 and anti-interleukin-23) (Torres et al., 2017; Roda et al., 2020; Cushing and Higgins, 2021; Nakase et al., 2021; Le Berre et al., 2023).

As the first biological agents, anti-tumor necrosis factor (anti-TNF) monoclonal antibodies, mainly including infliximab and adalimumab, have been widely used to treat moderate to severe IBD patients over the past two decades and have been proven effective and mature (Derkx et al., 1993; D'Haens and van Deventer, 2021; Rundquist et al., 2021). Unfortunately, 10%–40% of patients fail to respond favorably to the anti-TNF therapies for the first time, which is called primary non-response (PNR) (Ben-Horin et al., 2014; Papamichael et al., 2015; Sabino et al., 2019; Marsal et al., 2022). Moreover, secondary loss of response (LOR) is observed in 23%–50% of IBD patients with an initial clinical benefit, leading to dose-intensification or discontinuation of infliximab and adalimumab (Ben-Horin et al., 2014; Roda et al., 2016; Papamichael and Cheifetz, 2019; Sabino et al., 2019; Marsal et al., 2022).

Several factors contribute to PNR and LOR including disease characteristics (phenotype, location, severity), treatment strategies (dosing regimen), and drug problems (pharmacokinetics, pharmacodynamics, immunogenicity) (Ding et al., 2016). Among these reasons, immunogenicity failure, characterized by low or undetectable anti-TNF drug concentrations with high levels of anti-drug antibodies (ADAs), is considered one of the dominant factors (Moss et al., 2013; Ben-Horin et al., 2014; Ding et al., 2016; Vermeire et al., 2018). The formation of ADAs can impact the pharmacodynamics and pharmacokinetics of the anti-TNF drug, thereby reducing this drug’s efficacy and causing failures of disease remission (Moss et al., 2013; Lázár-Molnár and Delgado, 2016; Vaisman-Mentesh et al., 2020). The ADA can be divided into two major categories: neutralizing and non-neutralizing (Lázár-Molnár and Delgado, 2016; Vaisman-Mentesh et al., 2019). Neutralizing ADAs can directly block and interfere with the anti-TNF drug’s ability to bind TNF, decreasing or eliminating its ability to inhibit TNF-mediated signaling; non-neutralizing ADAs do not directly affect TNF binding but may compromise therapeutic efficacy through the formation of immune complexes and subsequent enhanced clearance of the drug from the circulation (Bendtzen, 2015; Lázár-Molnár and Delgado, 2016). According to a previous study, ADAs elicited in IBD patients treated with adalimumab or infliximab were predominantly neutralizing ADAs (van Schouwenburg et al., 2013).

Numerous risk factors have been identified for the development of ADA, including genetic predisposition, formation of drug-target complexes, combination therapy with immunomodulators, and antibiotic use differentially (Vermeire et al., 2018; Demase et al., 2023; Zhu et al., 2023). Concomitant treatment of anti-TNF drugs with thiopurines or methotrexate has been reported to improve the pharmacokinetics of anti-TNF agents to increase drug levels and reduce the formation of ADAs (Ungar et al., 2014; Ungar et al., 2017; Roblin et al., 2020; Bots et al., 2021; Demase et al., 2023; Yarur et al., 2023). However, there are safety concerns about the long-term concomitant use of immunomodulators, which are associated with serious adverse events including leukopenia, opportunistic infections, lymphoma, or other malignancies (Lemaitre et al., 2017; Kirchgesner et al., 2018; Luber et al., 2019). Therefore, the exploration of other strategies to reduce ADA formation and increase the drug response rate remains crucial in anti-TNF therapy of IBD.

Antibiotic use can not only treat infectious diseases effectively, but also result in alterations in the composition of intestinal microbiota (Becattini et al., 2016; Hagan et al., 2019). In recent years, more and more researchers have highlighted the role of intestinal microbial composition in the response to anti-TNF therapy (Bazin et al., 2018; Estevinho et al., 2020). Furthermore, a recent study from the Israeli IBD research nucleus revealed that the risk of ADA development can be increased in IBD patients exposed to cephalosporins or penicillin with β-lactamase inhibitors before or during anti-TNF therapy, while the treatment with fluoroquinolones or macrolides can possibly minimize the risk (Gorelik et al., 2022). This suggests that the use of specific antibiotic classes may potentially increase or reduce the risk of ADA formation during anti-TNF therapy through the specific alterations of the intestinal microbiome.

However, few studies assessed the effects of various antibiotic classes on ADA formation during anti-TNF therapy in Chinese IBD patients. Therefore, this study aimed to evaluate the potential effect of antibiotic treatment on immunogenicity to anti-TNF therapy in Chinese patients with IBD.

## 2 Materials and methods

### 2.1 Study population

IBD patients in this study were enrolled in the Department of Gastroenterology and Hepatology, West China Hospital (Sichuan, China) from January 2018 to October 2023.

IBD patients should be diagnosed based on the diagnostic criteria from the Chinese consensus on the diagnosis and treatment of inflammatory bowel disease (2018, Beijing). Only the IBD patients who were initially treated with anti-TNF therapy (infliximab or adalimumab) after January 2018 and who were reviewed with available ADA levels before October 2023 could be included in the study.

The study excluded patients who used other biological drugs within 6 months before anti-TNF therapy; patients with a history of mental illness, other immune system or hematological system diseases, malignant tumors, or any other conditions considered as an interference with the result analysis of the research. Under normal circumstances in our study, following the initial use of anti-TNF drugs, the infliximab was given at week 2, week 6, and then every 8 weeks; and the adalimumab was given every 2 weeks. Therefore, IBD patients with poor compliance and incomplete clinical data were also excluded.

**FIGURE 1:**
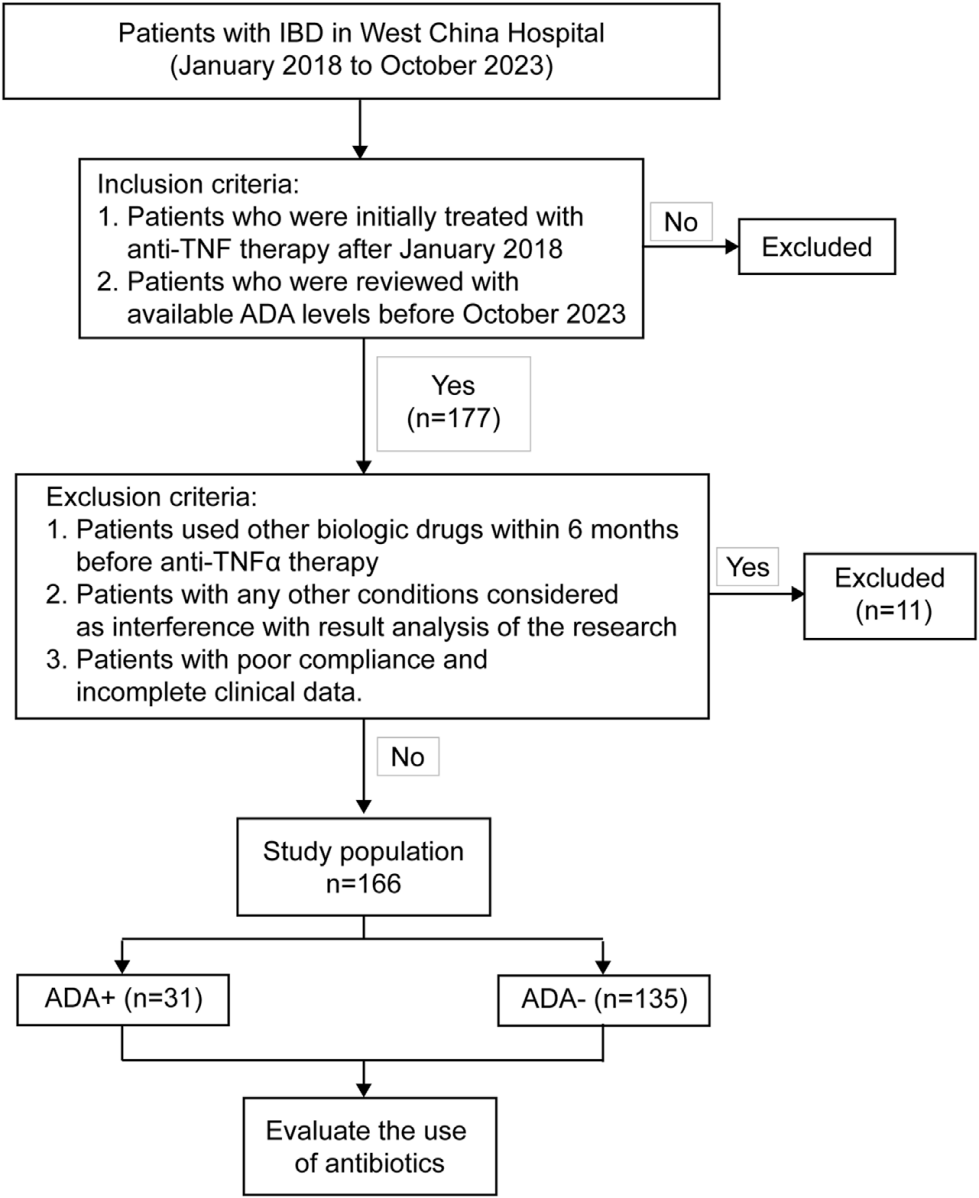
Flow chart of patient selection. 177 IBD patients received anti-TNF therapy (infliximab or adalimumab) with available ADA levels during January 2018 and October 2023.166 patients, who met the inclusion and exclusion criteria, finally entered the study.

Finally, we included 177 patients. Among these participants, 11 were excluded because they could not make a definitive diagnosis (n = 3), used anti-TNF therapy irregularly (n = 2), and could not find complete clinical data from the electronic medical records (n = 6). The remaining 166 IBD patients (149 CD and 17 UC) all met the study criteria and were enrolled in this retrospective analysis (Figure 1).

### 2.2 Baseline variables

A hospital information system was used to collect risk factors related to ADA development during anti-TNF therapy based on previous studies (Hässler et al., 2020; Bots et al., 2021; Zhu et al., 2023). At the first anti-TNF drug dispensation, we collected information about the patient’s sex, age at diagnosis, body mass index (BMI), IBD type, clinical parameters including disease location, disease behavior, related perianal diseases, disease activity, laboratory results [including hemoglobin (Hb), platelets (PLT), white blood cells (WBC), erythrocyte sedimentation rate (ESR), C-reactive protein (CRP) and albumin (ALB)], and prior use of biological agents (Table 1). Disease location and behavior for IBD were defined according to the Montreal classification (Satsangi et al., 2006). The Crohn’s disease activity index (CDAI) and the Mayo score were used to assess the disease activity of CD and UC (Best et al., 1976; D'Haens et al., 2007). At the end of the study, the data we collected included history of operation, smoking and drinking history, history of prior or concomitant IBD treatments like corticosteroids, 5-ASA, and immunosuppressants (such as azathioprine, methotrexate, and thalidomide), disease duration, duration of anti-TNF drug use, and the follow-up time (Table 1).

**TABLE 1:** Baseline characteristics of all IBD patients.

Baseline variables	Total IBD patients (N = 166)	IBD patients with negative ADA level (n = 135)	IBD patients with positive ADA level (n = 31)	*p*-Value
**Sex** [n (%)]				0.590
male	100 (60.2)	80 (59.3)	20 (64.5)	
female	66 (39.8)	55 (40.7)	11 (35.5)	
**Age** [years, median (IQR)]	26.0 (20.0, 31.0)	26.0 (20.0, 32.0)	25.0 (20.0, 28.0)	0.329
**BMI** [kg/m^2^, median (IQR)]	18.6 (16.9, 20.4)	18.6 (16.7, 20.2)	19.6 (16.9, 20.7)	0.260
**Related surgery** [n (%)]	110 (66.3)	88 (65.2)	22 (71.0)	0.539
**History of smoking** [n (%)]	18 (10.8)	13 (9.6)	5 (16.1)	0.466
**History of drinking** [n (%)]	13 (7.8)	11 (8.1)	2 (6.5)	1.000
**Disease duration** [months, median (IQR)]	62.0 (44.5, 99.3)	58.0 (40.0, 95.0)	79.0 (58.0, 106.0)	**0.009**
**Anti-TNF drug** [n (%)]				**<0.001**
Infliximab	108 (65.1)	79 (58.5)	29 (93.5)	
Adalimumab	58 (34.9)	56 (41.5)	2 (6.5)	
**IBD type** [n (%)]				0.271
CD	149 (89.8)	119 (88.1)	30 (96.8)	
UC	17 (10.2)	16 (11.9)	1 (3.2)	
**Age at diagnosis of CD^a^** [n (%)]				0.698
A1	18 (12.1)	13 (10.9)	5 (16.7)	
A2	114 (76.5)	92 (77.3)	22 (73.3)	
A3	17 (11.4)	14 (11.8)	3 (10.0)	
**Location of disease in CD patients^a^** [n (%)]				0.261
L1	15 (10.1)	14 (11.8)	1 (3.3)	
L2	26 (17.4)	19 (16.0)	7 (23.3)	
L2+3	1 (0.7)	1 (0.8)	0 (0.0)	
L2+4	2 (1.3)	1 (0.8)	1 (3.3)	
L3	94 (63.1)	77 (64.7)	17 (56.7)	
L3+4	6 (4.0)	4 (3.4)	2 (6.7)	
L3+4 + 1	1 (0.7)	1 (0.8)	0 (0.0)	
L4	4 (2.7)	2 (1.7)	2 (6.7)	
**Behavior of disease in CD patients** **^a^** [n (%)]				0.069
B1	42 (28.2)	38 (31.9)	4 (13.3)	
B2	42 (28.2)	28 (23.5)	14 (46.7)	
B2+3	18 (12.1)	16 (13.4)	2 (6.7)	
B3	45 (30.2)	35 (29.4)	10 (33.3)	
B3+4	2 (1.3)	2 (1.7)	0 (0.0)	
**Location of disease in UC patients** **^b^** [n (%)]				1.000
E1+2	2 (11.8)	2 (12.5)	0 (0.0)	
E2	2 (11.8)	2 (12.5)	0 (0.0)	
E3	13 (76.5)	12 (75.0)	1 (100.0)	
**Perianal disease** [n (%)]	75 (45.2)	59 (43.7)	16 (51.6)	0.425
**Disease activity** **^c^** [n (%)]				0.329
remission	32 (19.3)	28 (20.7)	4 (12.9)	
mild activity	31 (18.7)	22 (16.3)	9 (39.0)	
moderate activity	76 (45.8)	61 (45.2)	15 (48.4)	
severe activity	27 (16.3)	24 (17.8)	3 (9.7)	
**Laboratory results** [median (IQR)]				
Hb, g/L	123.0 (102.8, 138.3)	123.0 (102.0, 139.0)	120.0 (108.0, 138.0)	0.873
PLT, ×10^9^/L	277.0 (216.0, 365.3)	269.0 (216.0, 364.0)	304.0 (220.0, 377.0)	0.431
WBC, ×10^9^/L	6.0 (4.6, 7.6)	5.7 (4.5, 7.5)	6.4 (4.7, 8.1)	0.217
ESR, mm/h	36.5 (17.0, 56.5)	34.0 (16.0, 58.0)	43.0 (25.0, 56.0)	0.214
CRP, mg/L	8.2 (2.9, 26.4)	7.6 (2.9, 23.2)	13.4 (2.8, 30.7)	0.548
ALB, g/L	41.5 (37.5, 45.7)	41.6 (37.3, 45.8)	39.6 (37.5, 43.9)	0.547
**Prior use of biological agents** [n (%)]	27 (16.3)	22 (16.3)	5 (16.1)	0.982
**Prior/concomitant medications** [n (%)]				
Corticosteroid	75 (45.2)	61 (45.2)	14 (45.2)	0.998
5-ASA	135 (81.3)	107 (79.3)	28 (90.3)	0.154
Azathioprine	92 (55.4)	76 (56.3)	16 (51.6)	0.636
Thalidomide	28 (16.9)	23 (17.0)	5 (16.1)	0.903
Methotrexate	10 (6.0)	6 (4.4)	4 (12.9)	0.172
**Time from the first anti-TNF dispensation [days, median (IQR)]**				
Time to the first ADA test	252.5 (164.0, 437.5)	234.0 (160.0, 437.0)	306.0 (187.0, 509.0)	0.158
Time to the last anti-TNF dispensation	325.0 (173.5, 620.8)	332.0 (172.0, 610.0)	301.0 (184.0, 698.0)	0.998
Time to the end of follow-up	362.0 (191.3, 671.8)	362.0 (189.0, 666.0)	333.0 (193.0, 702.0)	0.906

Compare the baseline variables of IBD, patients with negative or positive ADA, levels. Continuous variables were presented with medians and IQR, and were analyzed by using the Mann-Whitney U test for data not normally distributed. Categorical variables were presented with absolute numbers and percentages, and were analyzed by using Pearson’s chi-square test or Fisher’s exact test. The results of variable comparisons that significantly differed between groups (*p* < 0.05) were highlighted in bold text.

IBD, inflammatory bowel disease; ADA, anti-drug antibodies; IQR, interquartile ranges; BMI, body mass index; CD, Crohn’s disease; UC, ulcerative colitis; A1, age≤16 years; A2, age 17–40 years; A3, age >40 years; L1, terminal ileum; L2, colon; L3, ileum colon; L4, upper gastrointestinal tract; B1, Non-narrow, non-fistula; B2, narrow; B3, fistula; E1+2, both proctitis and left-sided colitis; E2, left-sided colitis; E3, extensive colitis; Hb, hemoglobin; PLT, platelets; WBC, white blood cells; ESR, erythrocyte sedimentation rate; CRP, C-reactive protein; ALB, albumin; 5-ASA, 5-aminosalicylic acid; anti-TNF, anti-tumor necrosis factor.

The results of variable comparisons that significantly differed between groups (*p* < 0.05) were highlighted in bold text.

^a^ Applies to patients with CD.

^b^ Applies to patients with UC.

^c^ The disease activity of CD, was based on the Crohn’s disease activity index (CDAI) (CDAI <150, remission; 150–220, mild activity; 221–450, moderate activity; >450, severe activity). The disease activity of UC, was based on the Mayo score (score ≤2, remission; three to five, mild activity; 6–10, moderate activity; 11–12, severe activity).

### 2.3 Primary exposure and outcome

The follow-up period started at the first anti-TNF therapy after January 2018 and ended with the occurrence of the first positive ADA level measurement, or the last available negative ADA level measurement before the end of the study in October 2023.

At first, we selected the seven most frequently prescribed antibiotic classes as primary exposure variables, including penicillin, cephalosporins, β-lactam-β-lactamase inhibitor combinations (BL-BLIs), polypeptide, fluoroquinolones, nitroimidazoles, and antituberculotics. To further explore the persistent period of the influence of antibiotic use on ADA formation, antibiotic use was defined as any use of specific antibiotic classes during each period of time, including 12 months, 6 months, 3 months, or 1 month before the last ADA test at the end of the follow-up (Supplemental Table S1). During the 12 months prior to the last ADA test, we did not find any patient using penicillin, so we finally chose the other six antibiotic classes to analyze. Most antibiotic prescriptions were for approximately 1 week. In order not to miss any possible effect, we decided to define antibiotic use as using each antibiotic class no less than 3 days within a specific period of time before the ADA test.

The primary outcome was defined as the positive or negative serum ADA level at the endpoint of the follow-up period. Through fluorescence immunochromatography assays, serum ADA levels and drug concentration in IBD patients regularly treated with infliximab or adalimumab were measured close to the next infusion of anti-TNF preparations, which were detectable and quantifiable. Suzhou Herui Biotechnology Company carried out serum-free ADA levels and drug concentrations. After an acidic step to dissociate ADA-drug complexes, resulting in free ADAs and free drugs, the excess drugs or ADAs were then removed. Thus, the free ADAs or drugs could be captured by anti-TNF drugs or ADAs labeled by fluorescent microspheres on the reagent strips, and the detection of the free ADAs or drugs could be finally accomplished with the fluorescence immune analyzer. ADA level measurements were extracted as numeric values, and ADA levels at or above 30 ng/mL were defined as positive. The reference range of therapeutic serum drug concentration was above 3.0 μg/mL (infliximab) or 5.0 μg/mL (adalimumab).

### 2.4 Statistical analysis

A crude comparison of baseline characteristics between IBD patients with negative and positive ADA levels was performed using the Mann-Whitney U test for continuous variables and ordinal categorical variables, and Pearson’s chi-square test, Yates’s correction for continuity or Fisher’s exact test for unordered categorical variables. Continuous variables were presented with medians and interquartile ranges (IQR), and categorical variables were presented with absolute numbers and percentages. Among all the variables, there were a few missing values in BMI, CRP, ESR, ALB, and serum infliximab concentration, respectively accounting for 1.2%, 0.6%, 5.4%, 1.2%, and 1.2% of all patients. To avoid the loss of data, we interpolated serial mean values into these missing records.

To take into account that ADA occurrence was a dynamic event after the start of therapy, we decided to further perform survival analyses for the time-to-event data. The time-to-event (ADA positivity) data origin was defined as the time of initiation of anti-TNF drugs and the end of follow-up was defined as the date of the first positive ADA level or the last available negative ADA level. Univariable survival analysis of the association between the use of each antibiotic class and the risk of ADA development was performed using the Kaplan-Meier method and compared using the log-rank statistic. Using the same time origins and endpoints, we also performed a multivariable Cox proportional hazards model to evaluate the association between risk factors and ADA formation.

Baseline variables that showed a univariate relationship with the ADA formation (*p* < 0.2) or were considered clinically relevant based on prior studies entered into a multivariate Cox proportional hazard regression model (Roblin et al., 2020; Demase et al., 2023). Through univariate survival analysis, antibiotics that were used during a proper period of time for final inclusion in the multivariate analysis were also carefully chosen. To verify the assumption of proportional hazards in the Cox analysis, we used the Schoenfeld residual test showing that none of the variables were significant based on a *p*-value threshold of 0.05 (Supplemental Table S2).

During the different periods of time prior to the ADA test, many patients may be treated with more than one antibiotic class. Therefore, to evaluate possible synergistic and antagonistic effects of antibiotic combinations, we selected the antibiotic classes with significant effects on ADA development. For each pair from the selected combination, we analyzed the association between the ADA development and the use of both of the antibiotics from the pair, either or none by the Kaplan-Meier method and a log-rank statistic.

Multivariable adjusted hazard ratio (aHR) and 95% confidence intervals (CI) of variables were used to describe the risk of ADA development during anti-TNF therapy. All statistical analyses were performed by GraphPad Prism version 9.0, IBM SPSS Statistics version 27.0, and R version 4.3.2. All studies used 2-side tests, and the significant differences were documented as *p* < 0.05.

## 3 Results

### 3.1 Baseline characteristics of the study population

After a series of screenings, we finally included 166 IBD patients (149 CD, 17 UC) who were treated with infliximab or adalimumab and had available ADA levels during the treatment period to conduct data analysis (Figure 1). Most baseline factors, including sex, age, BMI, related surgery, history of smoking or drinking, IBD type and clinical classification based on Montreal classification, perianal disease, disease activity, various laboratory results, prior use of biological agents, prior or concomitant medications use, duration of anti-TNF drug use, and time from the first anti-TNF dispensation to first ADA test or the end of follow-up, showed no significant difference between IBD patients with negative or positive ADA levels (Table 1).

A total of 108 IBD patients (65.1%) were treated with infliximab, and the other 58 (34.9%) with adalimumab. And among the participants, 27 IBD patients (16.3%) had used biological agents before anti-TNF therapy. Overall, positive ADA levels were measured in 31 patients (18.7%). According to the baseline data, a total of 100 patients were male (60.2%), the median age at diagnosis of all participants was 26.0 (IQR: 20.0, 31.0) years old, and the median BMI was 18.6 (IQR: 16.9, 20.4) kg/m^2^. The median duration from the first anti-TNF dispensation to the first ADA test was 252.5 (IQR: 164.0, 437.5) days, the median duration of anti-TNF drug use was 325.0 (IQR:173.5, 620.8) days, and the median follow-up time started from anti-TNF treatment was 362.0 (IQR: 191.3, 671.8) days (Table 1).

Moreover, the duration of IBD seemed to be longer for patients with positive ADA levels (79.0 months) than those with negative ADA levels (58.0 months) (*p* = 0.009). Notably, patients who were treated with infliximab were more likely to develop ADAs than patients with adalimumab (*p* < 0.001) (Table 1).

### 3.2 The influence of antibiotics on ADA formation

**FIGURE 2:**
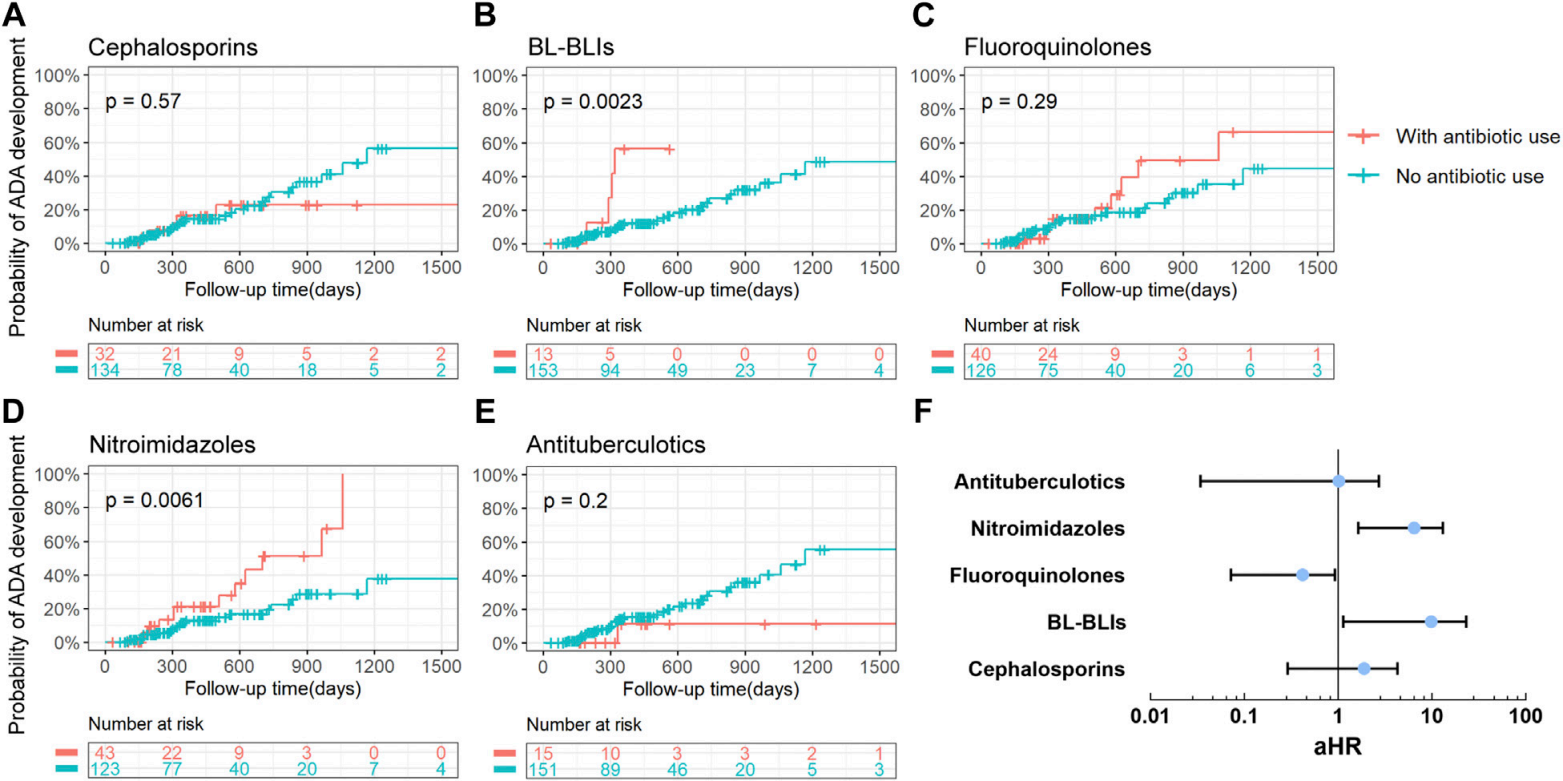
Analysis of the association between the use of antibiotics and the risk of ADA development. **(A–E)** Kaplan-Meier curves with risk tables of cumulative probability of ADA development for the use of different antibiotic classes during 12 months before the ADA test. Tick marks represented censored points and each plot was limited to the first 1500 days of follow-up time. A *p*-value for the log-rank test was presented in each plot. **(F)** The forest plot about multivariable-adjusted HRs (represented by blue points) for ADA development for the use of various antibiotic classes during 12 months before the ADA test. Adjusted HRs were presented on a log_10_ scale. Black lines represented 95% of CIs. ADA, anti-drug antibodies; BL-BLIs, β-lactam-β-lactamase inhibitor combinations; aHR, adjusted hazard ratio.

Among the six most frequently prescribed antibiotic classes, patients who used BL-BLIs or nitroimidazoles during 12 months prior to the last ADA test in our study showed an increased risk of ADA development in IBD patients treated with anti-TNF drugs (BL-BLIs: *p* = 0.002, nitroimidazoles: *p* = 0.006) (Figures 2B, D). And no other studied antibiotic classes were observed to be associated with ADA development in the univariable survival analysis (Figures 2A, C, E). Additionally, during other shorter periods of time (6 months, 3 months, and 1 month) before the ADA test, the use of nitroimidazoles also showed significant association with the development of the ADA (all *p* < 0.01) (Supplemental Table S1). And the most noteworthy difference was the loss of statistical significance of the effect of BL-BLI use during the shorter periods of time before the last ADA test on the ADA formation, probably due to the smaller sample size in this analysis (Supplemental Table S1). These results suggested that the persistent period of the influence of antibiotic use on ADA formation might extend up to 12 months prior to the ADA test. Thus, through univariate survival analysis, the antibiotic use within 12 months before the ADA test was finally chosen for inclusion in the multivariate Cox analysis.

Candidate variables with a univariate relationship (*p* < 0.2) and clinical relevance including disease duration, type of anti-TNF drugs, prior or concomitant use of 5-ASA and methotrexate, and other relevant variables that had been reported such as IBD type and azathioprine, were included in the multivariable model as confounders (Table 2) (Roblin et al., 2020; Demase et al., 2023). When entering these variables in the multivariate Cox proportional hazards model, an increased risk of ADA formation was noted in IBD patients who used BL-BLIs or nitroimidazoles during 12 months before the ADA test (BL-BLIs: HR = 5.143, 95%CI 1.136–23.270, *p* = 0.033; nitroimidazoles: HR = 4.635, 95%CI 1.641–13.089, *p* = 0.004), which were in agreement with the univariate survival analysis results. In contrast, we also found that fluoroquinolone use might reduce the risk of ADA formation (HR 0.258, 95% CI 0.072–0.924, *p* = 0.037) (Table 2; Figure 2F).

**TABLE 2:** Multivariable adjusted HRs for ADA development during anti-TNF therapy.

Variable	aHR (95% CI)	*p*-Value
**Antibiotic use during 12 months before the ADA test**		
Cephalosporins (yes vs. no)	1.111 (0.289, 4.278)	0.878
BL-BLIs (yes vs. no)	5.143 (1.136, 23.270)	**0.033**
Fluoroquinolones (yes vs. no)	0.258 (0.072, 0.924)	**0.037**
Nitroimidazoles (yes vs. no)	4.635 (1.641, 13.089)	**0.004**
Antituberculotics (yes vs. no)	0.306 (0.034, 2.721)	0.288
**Confounders**		
IBD type (CD vs. UC)	3.751 (0.471, 29.869)	0.212
Disease duration (per month increase)	1.003 (0.994, 1.013)	0.524
Anti-TNF drug (infliximab vs. adalimumab)	5.167 (1.072, 24.890)	**0.041**
5-ASA (yes vs. no)	2.871 (0.783, 10.532)	0.112
Azathioprine (yes vs. no)	0.270 (0.106, 0.682)	**0.006**
Methotrexate (yes vs. no)	2.042 (0.525, 7.939)	0.303

Data were shown for Cox proportional hazard models. Rows represented the covariates incorporated in the model and ordered as primary exposures (antibiotic use during 12 months before the ADA, test) and confounders. The results of comparisons showed significant differences with *p* < 0.05 were highlighted in bold text. ADA, anti-drug antibodies; aHR, adjusted hazard ratio; CI, confidence interval; BL-BLIs, β-lactam-β-lactamase inhibitor combinations; IBD, inflammatory bowel disease; CD, Crohn’s disease; UC, ulcerative colitis; anti-TNF, anti-tumor necrosis factor; 5-ASA, 5-aminosalicylic acid.

The results of comparisons showed significant differences with *p* < 0.05 were highlighted in bold text.

**FIGURE 3:**
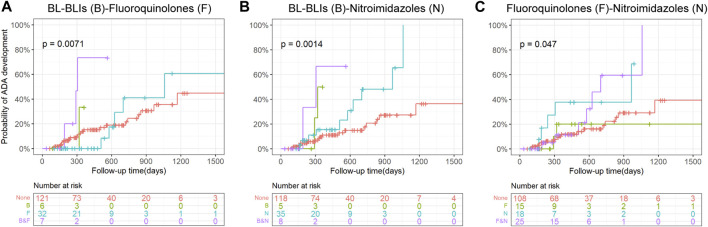
Kaplan-Meier curves with risk tables of cumulative probability of ADA development for the use of combinations of pairs from BL-BLIs, fluoroquinolones, and nitroimidazoles. Tick marks represent censoring. The plot is limited to the first 1500 days of follow-up. A *p*-value for the log-rank test is presented in each plot. ADA, anti-drug antibodies; BL-BLIs, β-lactam- β-lactamase inhibitor combinations.

Although a very small group of patients used both BL-BLIs and nitroimidazoles, they seemed to have a higher probability of ADA development compared with patients who used either alone (Figure 3B). When fluoroquinolones were used with BL-BLIs or nitroimidazoles, the mixed effects of combinations were similar to the alone effect of BL-BLI or nitroimidazole use (Figures 3A, C). These results might suggest that the BL-BLIs and nitroimidazoles might had some synergistic effect, whereas the effect of fluoroquinolones was possibly too weak to antagonize the increased risk of ADA formation. As a result, the absence of statistical significance of the effect of fluoroquinolone use on ADA formation in the univariate survival analysis might be explained by the potential effects of special antibiotic combinations.

Furthermore, we observed that, compared to adalimumab, IBD patients treated with infliximab during the follow-up time were associated with an increased risk of ADA formation (HR 5.167, 95% CI 1.072–24.890, *p* = 0.041). We also found a reduced hazard of ADA development in IBD patients who were treated with azathioprine (HR 0.270, 95%CI 0.106–0.682, *p* = 0.006) (Table 2).

### 3.3 Association between serum anti-TNF drug concentration and ADA formation

**FIGURE 4:**
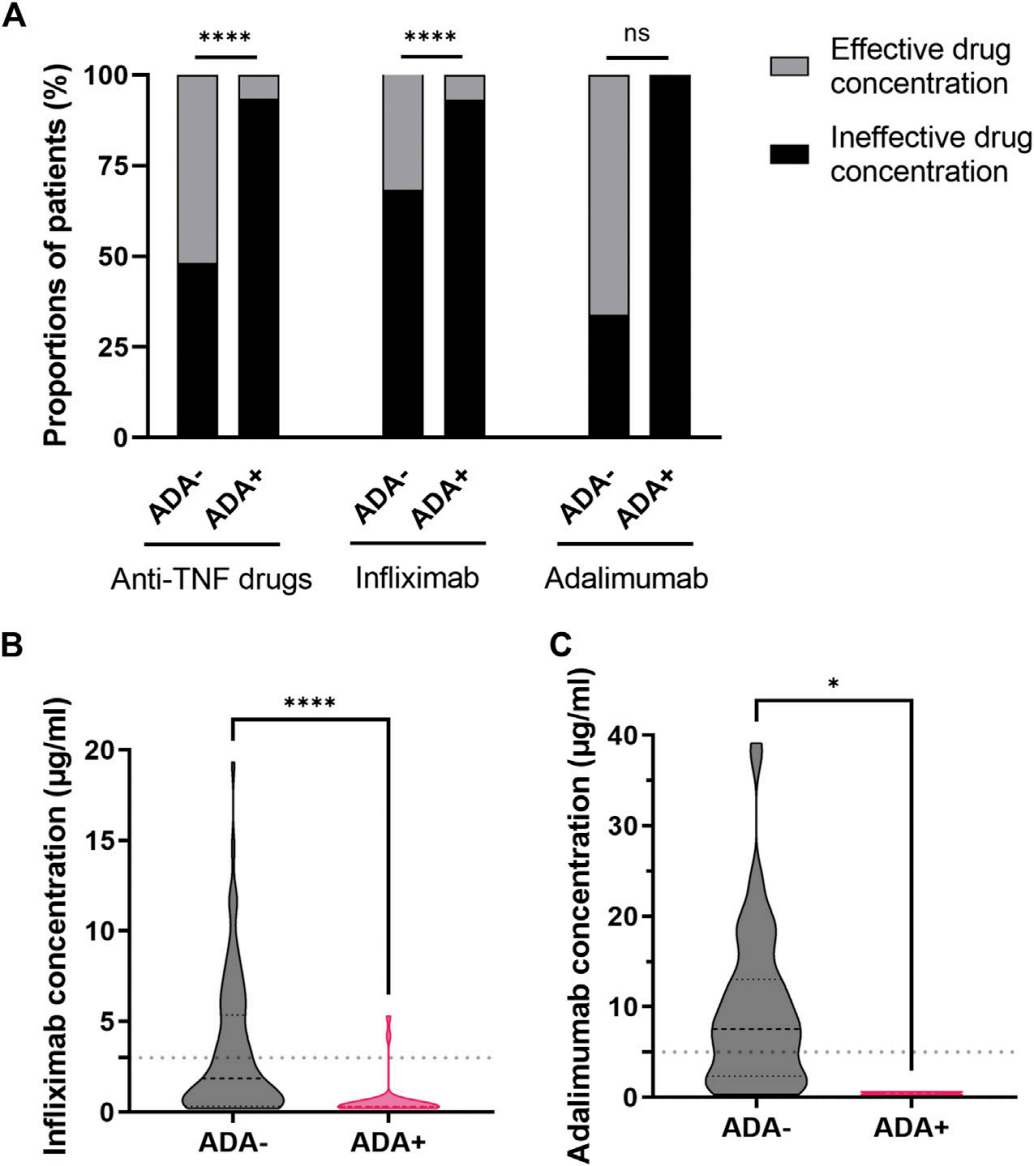
Association between serum anti-TNF drug concentration and ADA formation. **(A)** The proportions of patients with effective or ineffective drug (infliximab or adalimumab) concentration in patients with negative and positive ADA levels. The serum infliximab **(B)** or adalimumab **(C)** concentration in patients with negative and positive ADA levels. *p < 0.05, ****p < 0.0001 as determined by Pearson’s chi-square test or Fisher’s exact test **(A)** and Mann-Whitney U test **(B,C)**. The dotted lines represented the reference range of therapeutic drug (infliximab or adalimumab) concentration. ADA, anti-drug antibodies; anti-TNF, anti-tumor necrosis factor; ns, not significant.

In our study, the proportion of patients with effective drug concentration was 51.9% (69/135) in patients with negative ADA levels and 6.5% (2/31) in patients with positive ADA levels (Supplemental Table S3). Obviously, the proportions of patients with effective drug concentration (especially infliximab) in patients with positive ADA levels were significantly reduced (*p* < 0.0001) (Figure 4A). The results showed that the serum infliximab or adalimumab concentration in patients with positive ADA levels was generally lower than that in patients with negative ADA levels (infliximab: 0.30 vs. 1.85 μg/mL, *p* < 0.0001; adalimumab: 0.45 vs. 7.55 μg/mL, *p* = 0.0121) (Figures 4B,C).

## 4 Discussion

The primary findings of this study showed that the use of BL-BLIs or nitroimidazoles during 12 months prior to the ADA test can increase the risk of ADA formation in Chinese IBD patients. In contrast, fluoroquinolone use was associated with reduced immunogenicity.

Additionally, the analysis of patients’ serum anti-TNF drug concentration also pointed to a strong correlation between drug concentration and ADA formation. The negative association we found between serum anti-TNF drug concentration and ADA development concurs with previous research reports (Moss et al., 2013). Generally, higher drug concentrations were considered an effective outcome of treatment, while lower concentrations indicated the need to shorten dosing intervals, increase the drug dosage, or change the treatment method. The formation of the ADA is reported to lead to reducing serum drug levels by preventing anti-TNF drugs from binding to TNF or enhancing the clearance of the drug, leading to the failures of response to anti-TNF drugs and disease remission (Moss et al., 2013). Therefore, in order to raise the rate of response to anti-TNF drugs, it was important to explore the potential factors contributing to ADA formation.

Recently, the association of antibiotics with the development of the ADA has been reported in two studies, the participants of which were respectively from Europe and Israel (Hässler et al., 2020; Gorelik et al., 2022). However, few studies assessed the effect of various antibiotic classes on ADA formation during anti-TNF therapy in Chinese IBD patients.

In our study, we observed enhancing immunogenicity after the use of BL-BLIs or nitroimidazoles, and on the contrary, reduced immunogenicity with fluoroquinolone use. Previous studies revealed that gut microbiota could serve as potential biomarkers for predicting anti-TNF therapy response (Zhou et al., 2018; Estevinho et al., 2020). And the immunosuppressant effect of antibiotics was directly linked to changes in the gut microbiome in previous reports (Routy et al., 2018; Hagan et al., 2019). Therefore, the most likely explanation for the results would be the effect of antibiotics on the microbiome.

Several research projects reported a relatively higher abundance of *Clostridiales* in IBD patients responding to infliximab treatment at baseline or several months after initiation of anti-TNF therapy, which might suggest a positive association between *Clostridiales* and the response to anti-TNF drug in IBD patients (Kolho et al., 2015; Zhou et al., 2018; Sanchis-Artero et al., 2021; Park et al., 2022). A large systematic review suggested a decreased abundance of the order *Clostridiales* with the treatment of BL-BLIs, and an increase in some members of the *Clostridiales* order with fluoroquinolone use (Nel Van Zyl et al., 2022). Moreover, the main use of nitroimidazoles is infection treatment or prevention caused by anaerobic bacteria, which remains a good choice for colitis associated with *Clostridium difficile* (Dingsdag and Hunter, 2018). One study reported that pre-treatment with metronidazole (one of the nitroimidazoles) before fecal microbiota transplantation (FMT) administration to colitis animals could significantly reduce the relative abundance of *Clostridia* (*Clostridium XI* and *unclassified Clostridiaceae1*) (Strati et al., 2021). These results might suggest that the different effects of various antibiotics in our analysis, including increased immunogenicity in BL-BLI or nitroimidazole use with possible synergism between the two classes and decreased immunogenicity with the use of fluoroquinolones, could be probably explained by the specific dysbiosis caused by these antibiotics.

Furthermore, in a T-cell-dependent pathway, ADAs are generated when a T helper cell (Th) differentiates into a Th1 or Th2 phenotype (Vaisman-Mentesh et al., 2020). It has been reported that circulating infliximab-specific Th2 cells can be detected mainly in treated patients developing ADA (Vultaggio et al., 2016). Microbial therapy with the *Clostridiales* consortium has been shown to upregulate the induction of ROR-γt regulatory T (Treg) cells that were reported to play a critical role in suppressing Th2 cell responses (Abdel-Gadir et al., 2019; Stephen-Victor et al., 2020; Turner et al., 2020). Therefore, these study findings seemed to imply that, through inhibiting Th2 cell responses, *Clostridiales* species could possibly reduce the development of ADAs to improve patients’ response to the anti-TNF drugs, which was consistent with our results about the effects of various antibiotic classes on the immunogenicity. Additionally, the association between fluoroquinolones and reduced immunogenicity also concurred with the anti-inflammatory roles of suppression of the TNFα levels (Ogino et al., 2009). Thus, more detailed mechanisms of the effects of antibiotics on ADA formation during anti-TNF therapy need to be further investigated.

According to the study performed by the European consortium ABIRISK, antibiotic use could reduce the risk of ADA formation (Estevinho et al., 2020). Moreover, the Israeli conducted a clinical retrospective study of 1946 IBD patients and found that cephalosporins and penicillin-BLIs were associated with increased risk of ADA development, whereas fluoroquinolones and macrolides were associated with decreased risk, which partly contradicted our results (Gorelik et al., 2022). As is widely known, host lifestyle could affect gut microbiota a lot. The gut microbiota harbored by the Chinese population are different from those harbored by the population from other areas due to the differences in ethnicity and diet (Prideaux et al., 2013; Clements and R Carding, 2018; Qiu et al., 2020; Sugihara and Kamada, 2021). The different influence of various antibiotic classes on ADA formation can be explained by the considerably different intestinal microbiome communities and the different habits of antibiotic usage. Therefore, it is necessary to explore the influence of various antibiotic classes on ADA formation during anti-TNF therapy in Chinese IBD patients.

An additional observation was that the persistent period of the influence of antibiotic use on ADA formation might extend up to 12 months prior to the ADA test. Many studies have demonstrated that, while most of the intestinal microbial perturbations (both in composition and diversity) could return to baseline levels in relatively short periods of time (one to 3 months after antibiotic use), other small parts of the microbial dysbiosis might persist for up to 1 year or even longer after the antibiotic treatment (Lankelma et al., 2016; Haak et al., 2019; Nel Van Zyl et al., 2022). To further explore the persistent period of the influence of antibiotic use on ADA formation, antibiotic use was defined as any use of specific antibiotic classes during each period of time, including 12 months, 6 months, 3 months, or 1 month before the last ADA test. Ultimately, we found the effects of specific antibiotic classes on the ADA formation might last at least 12 months.

Our results also suggested that, compared to adalimumab, IBD patients treated with infliximab during the follow-up time were easier to form high concentrations of ADAs, which was in line with the previous studies. Previous studies have revealed that adalimumab, a fully human IgG1 monoclonal antibody binding with high affinity and specificity to membrane and soluble TNF, had shown lower immunogenicity and higher safety compared to infliximab, a human-murine chimeric monoclonal IgG1 antibody (Breedveld, 2000; Baert et al., 2003; Sandborn et al., 2007).

Many researches have demonstrated that combination therapy with immunosuppressants could increase anti-TNF drug concentration and decrease ADA formation (Garcês et al., 2013; Thomas et al., 2015). The analysis of clinical data in our study also suggested the immunogenicity-reducing effect of prior or concomitant azathioprine use with anti-TNF therapy. However, our study did not find an association between methotrexate and ADA formation. And the limited sample size might be a key factor in the different results. In our clinical data, only a small group of IBD patients treated with anti-TNF drugs had used methotrexate, which could not provide sufficient statistical power to determine its effect on ADA formation. Perhaps in a larger sample study, we can find significant associations between these immunosuppressants and ADA development.

Despite these promising results, some limitations of our retrospective study should be mentioned. One shortcoming of fluorescence immunochromatography assays detecting ADAs is that they do not identify functional neutralizing or non-neutralizing ADAs. Although non-neutralizing ADAs may indirectly reduce a drug’s efficacy, neutralizing ADAs are generally considered more important in a clinical setting because of their direct effect on the drug’s biological activity (Bendtzen, 2015; Lázár-Molnár and Delgado, 2016). Therefore, the accurate measurement of neutralizing ADAs may lead to a more precise understanding of the risk factors and relevant mechanisms of treatment failures. Moreover, previous studies have reported the phenomenon of transient ADAs, which are usually of low titer, might disappear on subsequent infusions with little clinical effect (Ben-Horin and Chowers, 2014). Nevertheless, all the ADA levels we collected in our study could not perfectly distinguish the transient or sustained ADAs of every patient, because some patients with the first positive ADA level would prefer to switch treatment medications. Additionally, apart from the analysis of the persistent period of the influence of antibiotic use on ADA formation, the number of repetitions, the duration, and the dosage of the antibiotic use might influence the drug effects on ADA formation, which should be carefully considered in future related studies.

Notably, through many literature reviews, we just put forward a hypothesis about the possible mechanisms of the effects of antibiotics on ADA formation, without any clear evidence to directly support it. Prospective cohort studies with larger sample size, more detailed data and human gut microbiome at the starting and ending point of follow-up time, and multiple experimental studies are therefore needed to further explore the specific mechanism of antibiotic effects on immunogenicity. In a word, due to these limitations and the lack of replication studies related to the antibiotic effects on ADA formation in Chinese IBD patients, the results of our study are just for reference, and more relevant studies are required to further confirm our findings.

In conclusion, our study indicated that the use of BL-BLIs and nitroimidazoles within 12 months prior to the ADA test might increase the risk of ADA formation during anti-TNF therapy in Chinese IBD patients, while the treatment with fluoroquinolones could probably reduce such risk. The results indicated the association of various antibiotic classes with ADA development, which might suggest more careful use of antibiotics during anti-TNF treatment.

## Data Availability

The raw data supporting the conclusion of this article will be made available by the authors, without undue reservation.
