# Supplementary material for: Antibiotics influence the risk of anti-drug antibody formation during anti-TNF therapy in Chinese inflammatory bowel disease patients
Source: Front Pharmacol. 2024 Apr 9;15:1360835. doi: 10.3389/fphar.2024.1360835 (PMC11035825; doi:10.3389/fphar.2024.1360835)
Supplement: Supplementary file 1 [file Table1.docx]

Supplementary Material

# Supplementary Tables

**Supplementary Table 1.** Univariable survival analysis of the association between the use of antibiotics and the risk of ADA development. The analysis was performed using the Kaplan-Meier method and compared using the log-rank statistic. Antibiotic use was defined as any use of specific antibiotic classes during each period of time (12 months, 6 months, 3 months, or 1 month before the last ADA test). The results of comparisons showed significant differences with p<0.05 were highlighted in bold text.

| Antibiotic use | Total  IBD patients  (N=166) | IBD patients  with negative  ADA level  (n=135) | | IBD patients  with positive  ADA level  (n=31) | P value |
| --- | --- | --- | --- | --- | --- |
| During 12 months before the ADA test [n (%)] | | |  | |  |
| Cephalosporins | 32 (19.3) | 27 (20.0) | | 5 (16.1) | 0.573 |
| BL-BLIs | 13 (7.8) | 9 (6.7) | | 4 (12.9) | **0.002** |
| Polypeptides | 1 (0.6) | 1 (0.7) | | 0 (0.0) | -* |
| Fluoroquinolones | 40 (24.1) | 31 (23.0) | | 9 (29.0) | 0.292 |
| Nitroimidazoles | 43 (25.9) | 31 (23.0) | | 12 (38.7) | **0.006** |
| Antituberculotics | 15 (9.0) | 14 (10.4) | | 1 (3.2) | 0.195 |
| During 6 months before the ADA test [n (%)] | | |  | |  |
| Cephalosporins | 18 (10.8) | 14 (10.4) | | 4 (12.9) | 0.479 |
| BL-BLIs | 8 (4.8) | 6 (4.4) | | 2 (6.5) | 0.064 |
| Polypeptides | 1 (0.6) | 1 (0.7) | | 0 (0.0) | -* |
| Fluoroquinolones | 26 (15.7) | 20 (14.8) | | 6 (19.4) | 0.530 |
| Nitroimidazoles | 30 (18.1) | 19 (14.1) | | 11 (35.5) | **＜0.001** |
| Antituberculotics | 9 (5.4) | 8 (5.9) | | 1 (3.2) | 0.852 |
| During 3 months before the ADA test [n (%)] | | |  | |  |
| Cephalosporins | 13 (7.8) | 10 (7.4) | | 3 (9.7) | 0.737 |
| BL-BLIs | 5 (3.0) | 4 (3.0) | | 1 (3.2) | 0.463 |
| Polypeptides | 0 (0.0) | 0 (0.0) | | 0 (0.0) | -* |
| Fluoroquinolones | 13 (7.8) | 9 (6.7) | | 4 (12.9) | 0.454 |
| Nitroimidazoles | 13 (7.8) | 4 (3.0) | | 9 (29.0) | **＜0.001** |
| Antituberculotics | 4 (2.4) | 3 (2.2) | | 1 (3.2) | 0.428 |
| During 1 month before the ADA test [n (%)] | | |  | |  |
| Cephalosporins | 6 (3.6) | 5 (3.7) | | 1 (3.2) | 0.590 |
| BL-BLIs | 3 (1.8) | 3 (2.2) | | 0 (0.0) | 0.676 |
| Polypeptides | 0 (0.0) | 0 (0.0) | | 0 (0.0) | -* |
| Fluoroquinolones | 4 (2.4) | 3 (2.2) | | 1 (3.2) | 0.656 |
| Nitroimidazoles | 9 (5.4) | 3 (2.2) | | 6 (19.4) | **0.002** |
| Antituberculotics | 3 (1.8) | 2 (1.5) | | 1 (3.2) | 0.371 |

IBD, inflammatory bowel disease; ADA, anti-drug antibodies; BL-BLIs, β-lactam-β-lactamase inhibitor combinations.

*Antibiotics that less than 1% of the IBD patients used were excluded from the univariable survival analysis.

**Supplementary Table 2.** Variables included in the multivariable analysis and calculated Schoenfeld residuals for the Cox regression model.

| Variable | Chi square | Degrees of freedom | P value | |
| --- | --- | --- | --- | --- |
| Cephalosporins | 0.06 | 1 | 0.80 | |
| BL-BLIs | 0.83 | 1 | 0.36 | |
| Fluoroquinolones | 3.83 | 1 | 0.05 | |
| Nitroimidazoles | 2.31 | 1 | 0.13 | |
| Antituberculotics | 0.10 | 1 | 0.75 | |
| Crohn’s Disease | 1.05 | 1 | 0.31 |  |
| Disease duration (per month increase) | 0.20 | 1 | 0.65 |  |
| Infliximab | 0.77 | 1 | 0.38 |  |
| 5-ASA | 0.00 | 1 | 0.99 |  |
| Azathioprine | 0.23 | 1 | 0.63 |  |
| Methotrexate | 0.00 | 1 | 0.96 |  |
| Global | 12.5 | 11 | 0.33 |  |

BL-BLIs, β-lactam-β-lactamase inhibitor combinations; 5-ASA, 5-aminosalicylic acid.

**Supplementary table 3.** Association between serum anti-TNF drug concentration and ADA formation. Continuous variables were presented with medians and IQR, and were analyzed by using Mann-Whitney U test. Categorical variables were presented with absolute number and percentages, and were analyzed by using Fisher’s exact test. The results of variable comparisons that significantly differed between groups (p<0.05) were highlighted in bold text.

| Variable | IBD patients with negative ADA level (n=135) | IBD patients with positive ADA level (n=31) | P value |
| --- | --- | --- | --- |
| Effective drug concentration* [n (%)] | 69 (51.9) | 2 (6.5) | **<0.0001** |
| Effective infliximab concentration*  [n (%)] | 32 (41.6) | 2 (6.9) | **<0.0001** |
| Effective adalimumab concentration  [n (%)] | 37 (66.1) | 0 (0.0) | 0.1270 |
| Serum infliximab concentration*  [μg/ml, median (IQR)] | 1.85 (0.33, 5.35) | 0.30 (0.30, 0.30) | **<0.0001** |
| Serum adalimumab concentration  [μg/ml, median (IQR)] | 7.55 (2.35, 13.05) | 0.45 (0.30, 0.60) | **0.0121** |

IBD, inflammatory bowel disease; ADA, anti-drug antibodies; IQR, interquartile ranges.

*The values of serum infliximab concentration from two patients (1.2%) with negative ADA levels were missing. To avoid the loss of data, we interpolated serial mean values of infliximab concentration into these missing records.
